# Daily mindfulness, negative affect, and eating behaviors in adolescents at risk for excess weight gain

**DOI:** 10.1002/eat.23981

**Published:** 2023-05-09

**Authors:** Reagan L Miller, Ruth M. Bernstein, Natalia Sanchez, Ana M. Gutierrez-Colina, Elizabeth B. Ruzicka, Christopher Bell, Sarah A. Johnson, Lauren B. Shomaker, Rachel G. Lucas-Thompson

**Affiliations:** 1 Department of Human Development & Family Studies, College of Health & Human Sciences, Colorado State University, Fort Collins, Colorado, USA; 2 Bariatric & Weight Management Center, Wake Forest School of Medicine, Winston-Salem, North Carolina, USA; 3 Department of Healthy & Exercise Science, College of Health & Human Sciences, Colorado State University, Fort Collins, Colorado, USA; 4 Department of Food Science & Human Nutrition, College of Health & Human Sciences, Colorado State University, Fort Collins, Colorado, USA; 5 Colorado School of Public Health, Fort Collins, Colorado, USA

**Keywords:** adolescent, affect, body mass index, loss of control eating, mindfulness, obesity, repeated-measures

## Abstract

**Objective:**

Loss-of-control and overeating are common in adolescents with high body mass index (BMI). Mindfulness may affect negative affect, and both may relate to loss-of-control and overeating. Yet, there is limited understanding of these associations in adolescents’ daily lives.

**Methods:**

Forty-five adolescents (77% female; *M*_age_ = 14.4 years, *SD*_age_ = 1.7 years) with high weight (92% with BMI [kg/m^2^] ≥85th percentile for age/sex) provided daily, repeated measurements of mindfulness, negative affect, loss-of-control, and overeating for ~7 days (*M* = 5.6 days; range = 1–13). Multilevel mixed modeling was conducted to test within-person (intraindividual) and between-person (interindividual) associations for the same-day (concurrent) and next-day (time-ordered/prospective).

**Results:**

There were within-person and between-person associations of higher mindfulness with lower negative affect on the same-day and next-day. Greater between-person mindfulness related to lower odds of adolescents’ loss-of-control occurrence (same-day) and conversely, more perceived control over eating (same-day and next-day). Greater within-person mindfulness related to less odds of next-day overeating.

**Discussion:**

Dynamic relations exist among mindfulness, negative affect, and eating in adolescents at-risk for excess weight gain. Mindfulness may be an important element to consider in loss-of-control and overeating. Future work using momentary-data within an experimental design would help disentangle the intraindividual effects of increasing mindfulness/decreasing negative affect on disordered eating.

**Public Significance:**

Loss-of-control and overeating are common in teenagers with high weight. Greater mindfulness—present-moment, non-judgmental attention—and less negative emotions may relate to healthier eating, but we do not know how these processes play out in teenagers’ daily lives. Addressing this knowledge gap, the current findings showed that greater daily mindfulness, but not negative affect, related to less loss-of-control/overeating, suggesting the importance of mindfulness for eating patterns in teenagers’ daily lives.

## 1 | INTRODUCTION

Loss-of-control and overeating are common in adolescents with high weight (Ackard et al., 2003; He et al., 2017), and can contribute to the onset of binge eating (Tanofsky-Kraff et al., 2011). Extant theory and research suggest that mindfulness, a trait-like characteristic of paying attention to the present moment with non-judgment (Kabat-Zinn, 2003), and negative affect are important when characterizing risk for loss-of-control/overeating (Berg et al., 2015; Ouwens et al., 2015). Most research has not focused on adolescence, despite it being a time of high-risk for developing loss-of-control/overeating (Goldschmidt et al., 2016; Swanson et al., 2011). Further, most adolescent investigations have described between-person (interindividual) effects of processes occurring within the same day, without investigating within-person (intraindividual) effects. Likewise, most daily process studies report same-day (concurrent) effects, with limited tests of next-day (time-ordered/prospective) associations from one day to the next (Snippe et al., 2015), which can lend more information for formulating theories of putative mechanisms.

Monitor and acceptance (Lindsay & Creswell, 2017) and affect regulation theories (Hawkins & Clements, 1984) suggest inverse associations of mindfulness and negative affect with loss-of-control/overeating. Monitor and acceptance theory (Lindsay & Creswell, 2017) posits that mindfulness promotes healthier behaviors by increasing attention/acceptance of present-moment experiences. Relatedly, mindfulness may foster interoceptive awareness, sensing/integrating of internal processes, which may help to regulate and improve negative affect as well as behaviors like food intake (Füstös et al., 2013; Hanley et al., 2017). Alternatively, negative affect may degrade mindfulness (Karl & Fischer, 2022). According to affect regulation theory, loss-of-control (inability to stop eating over any amount of food) and overeating (consumption of a large amount without loss-of-control; van Strien et al., 2012) occurs, in part, as an attempt to regulate negative mood (Egbert et al., 2022; Elliott et al., 2010).

Consistent with theoretical frameworks, higher daily mindfulness is related to lower same-day (Brockman et al., 2017) and next-day (Snippe et al., 2015, 2017) negative affect among adults, suggesting mindfulness may have both immediate and short-term/delayed effects on emotional processes. Day-to-day mindfulness and negative affect among adolescents are not well understood. Instead, cross-sectional evidence suggests that greater adolescent mindfulness relates to lower negative affect (Tumminia et al., 2020), less test-meal eating in the absence of hunger (Annameier et al., 2018), and lower odds of binge-eating (Pivarunas et al., 2015). Thus, it is possible that greater daily mindfulness may result in lower negative affect and loss-of-control/overeating among adolescents.

There is more research examining the role of negative affect in loss-of-control and emotional eating in adolescents. Adolescents’ greater state negative affect prior to a test-meal related to greater intake of snacks/desserts and carbohydrates (Ranzenhofer et al., 2013). Qualitatively, older adolescents expressed that a buildup of stress/emotions can influence their desire to “eat more” and feel as though they cannot resist food (Bennett et al., 2013, p. 189). However, in ecological momentary assessment (EMA) studies, negative affect has not predicted adolescents’ loss-of-control (Goldschmidt et al., 2018; Ranzenhofer et al., 2014). One possibility is that factors that relate to emotion regulation, like mindfulness, may be more salient for day-to-day fluctuations in loss-of-control/overeating (Walenda et al., 2021). Further, as most studies have tested same-day/concurrent effects at the between-person level, investigations of time-ordered/prospective and within-person effects are necessary to shed light on mindfulness/negative affect as possible precipitants of loss-of-control/overeating.

This study examined if: (1) within-person and between-person mindfulness were associated with same-day and next-day negative affect, and vice versa, and (2) within-person and between-person mindfulness and negative affect were associated with same-day and next-day loss-of-control/overeating. We hypothesized that greater daily mindfulness would be associated with lower same-day and next-day negative affect, within/between adolescents. Also, greater negative affect was expected to be associated with lower same-day and next-day mindfulness, within/between adolescents. Additionally, we hypothesized that greater mindfulness and lower negative affect, both within/between adolescents, would relate to less odds of same-day and next-day loss-of-control/overeating and conversely, greater perceived control over eating.

## 2 | METHODS

### 2.1 | Participants

Adolescents (*N* = 45) were recruited from the baseline phase of two trials piloting group programs to prevent unhealthy weight gain or lower type 2 diabetes risk (Table 1). Adolescents were 12–17 years and in good general health. In one trial (NCT03085160; Shomaker et al., 2019), participants were males and females at-risk for excess weight gain based on high body mass index (BMI; ≥70th percentile for age/sex) or family history of obesity (parental BMI ≥30 kg/m^2^). In the other (NCT02218138; Shomaker et al., 2017), participants were females, had BMI ≥85th percentile, diabetes family history, and mild-to-moderate depression symptoms. There were no differences in race/ethnicity, age, and BMI-*z* by study (*p*-values >.28), but sex varied by design.

### 2.2 | Procedure

Procedures of this repeated-measure design were approved by Colorado State University’s Institutional Review Board. Written consent/assent were obtained before procedures began. At baseline, adolescents received education on loss-of-control/overeating and instructions for answering an online questionnaire once per day in the evening for ≥7 days (up to 14 days for flexibility) through SurveyMonkey. Adolescents received ~$3 per survey. All measures were used in prior daily-level research, with evidence for reliability and ties to relevant constructs (Lucas-Thompson et al., 2021; Ranzenhofer et al., 2014), or were modified for daily use from reliable, valid measures (Brown & Ryan, 2003; Fairburn et al., 1993).

### 2.3 | Measures

#### Mindfulness.

Participants completed the 5-item Mindful Attention and Awareness Scale (MAAS) and one item about snacking without awareness from the 15-item MAAS (Brown & Ryan, 2003). Participants rated extent to which they had experienced each statement on a scale from 1 (not at all) to 7 (almost always). All items were reverse coded and averaged (Ω_b_ = .93; Ω_w_ = .68).

#### Negative affect.

Participants responded to 5-items on the Positive and Negative Affect Schedule (Watson et al., 1988). Adolescents rated how much they felt distressed, upset, scared, afraid, and nervous using a scale from 1 (none) to 5 (extremely). Mean scores were calculated (Ω_b_ = .95; Ω_w_ = .69).

#### Loss-of-control.

Presence (vs. absence) of loss-of-control was assessed through endorsement of, “Since the last time you completed this form, have you lost control over your eating?” If the answer was “yes,” continuous level of control over eating was rated from 1 (none) to 5 (a lot). Lower scores indicated less control over eating during a loss-of-control episode.

#### Overeating only.

In line with the literature (Fairburn et al., 1993; Ranzenhofer et al., 2014), overeating was assessed through the endorsement of, “Since the last time you completed this form, have you eaten too much food at one time?” Individuals who responded “yes” and did not endorse loss-of-control were considered to have an overeating episode.

#### Body composition.

Fasting weight was measured to the nearest 0.1 kg using a calibrated Etekcity digital scale. Height was recorded as the average of three measurements using a calibrated wall HM 200P Portstad stadiometer. BMI/BMI-*z* were calculated according to CDC 2000 standards (Kuczmarski et al., 2002).

### 2.4 | Data analysis

Point-biserial correlations and Fisher’s exact tests were used to investigate if 80% compliance (Lischetzke & Könen, 2020) was associated with demographic variables. For next-day analyses, lagged variables were created. To investigate within-person vs. between-person variation in outcomes, we examined intraclass correlation coefficients (ICCs) from an intercept-only model (Table 2). When ICCs are >.05, multilevel modeling is used to account for nesting (Kreft & De Leeuw, 1998). We conducted mixed-effect models in Stata 15 with random intercepts, which are robust at handling repeated measurements and varying numbers of observations per person (Hedeker & Nordgren, 2013; Leckie, 2014). Mixed-effects logistic models were used to investigate presence/absence of loss-of-control and overeating. Full likelihood estimation was used to handle missing data. Within-person associations were modeled by disaggregating individual-level variability from average, between-level differences. Mindfulness and negative affect were included as predictors in the same model when investigating same-day (concurrent) and next-day (prospective) eating. Next-day analyses controlled for same-day processes.

## 3 | RESULTS

Adolescents provided 259 days of observations (*M* = 5.6 days; range = 1–13); most were completed in the evening (Median = 8:35 pm). Eighty-percent compliance was not associated with race/ethnicity, BMI-*z*, or age (*p*-values >.08).

There were inverse associations of mindfulness within-person (*B* = −.28, *SE* = .06, *p* < .001) and between-person (*B* = −.22, *SE* = .07, *p* < .01) with same-day negative affect. Mindfulness also was associated with next-day negative affect between-person (*B* = −.13, *SE* = .06, *p* = .03), but not within-person (*B* = .08, *SE* = .09, *p* = .38). In contrast, negative affect was associated with same-day mindfulness between-person (*B* = −.75, *SE* = .25, *p* = .003) and within-person (*B* = −.33, *SE* = .07, *p* < .001), but not next-day (*p*-values >.07).

Between-person mindfulness was inversely associated with same-day loss-of-control (OR = .31, *SE* = .15, *p* = .02). Between-person mindfulness also was associated with greater same-day (*B* = .55, *SE* = .24, *p* < .01) and next-day (*B* = .57, *SE* = .21, *p* < .01) degree of control over eating during a loss-of-control episode. Within-person mindfulness was inversely associated with next-day overeating (OR = .23, *SE* = .15, *p* = .03). There were no other associations between negative affect nor mindfulness with same-day/next-day loss-of-control or overeating (*p*-values >.09).

## 4 | DISCUSSION

In line with previous research (Snippe et al., 2015), there were both within-person (intraindividual) and between-person (interindividual) associations among mindfulness and negative affect on the same-day and from one day to the next-day. Specifically, same-day intraindividual and interindividual variability in adolescents’ higher mindfulness related to their lower negative affect. However, on the next-day, associations were only observed interindividually. In addition, there were no significant associations between negative affect (intraindividually/interindividually) and next-day mindfulness. These results suggest that variations in how mindful an adolescent is relative to their own average (i.e., within-person) may be most related to negative affect concurrently within the same day. Conversely, overall mindfulness (i.e., between-persons) may have time-ordered effects on emotional processes the following day. One explanation for these findings could relate to the possibility that greater intraindividual mindfulness may not be as potent the following day without adequate mindfulness-training (Lucas-Thompson et al., 2021). Going forward, this notion should be investigated among additional samples of adolescents to determine time-ordered effects between mindfulness and negative affect.

Unexpectedly, there were more significant associations between mindfulness, loss-of-control, and control over eating during a loss-of-control episode between-person, the same-day and next-day, rather than within-person. Specifically, greater overall mindfulness was related to lower odds of having loss-of-control and greater control over one’s eating during a loss-of-control episode. Within-person mindfulness was only associated with overeating. No associations were observed between negative affect and eating behaviors. These results are in line with other between-person studies (Pivarunas et al., 2015; Shomaker et al., 2018) and youth EMA research (Goldschmidt et al., 2018; Ranzenhofer et al., 2014). They also highlight how mindfulness, as opposed to negative affect, may be especially salient for efforts to improve adolescents’ eating behaviors. One explanation for these findings relates to the notion that increased mindful attention may improve awareness/responsiveness to hunger/satiety cues, which may, in turn, reduce loss-of-control (Bennett & Latner, 2022). Lack of within-person findings may be related to our daily-level measurement approach; future research should incorporate momentary-level data throughout the day. Additionally, in a larger sample, tests of mediation (e.g., mindfulness → negative affect → eating behavior) and moderation (e.g., buffering effects of mindfulness) would help to clarify associations among mindfulness, negative affect, and eating more fully.

Although this study contributes meaningful information about connections among mindfulness, negative affect, and loss-of-control/overeating among adolescents in their daily lives, the sample was a small, convenience sample of adolescents at-risk for excess weight gain, with limited racial/ethnic heterogeneity. Although we had adequate power to detect small-to-medium effects (Arend & Schäfer, 2019), findings should be replicated among a larger and more heterogeneous sample. Additionally, there are several possible methodological limitations including a lack of randomization during survey delivery and use of binary measurements. Adolescents were trained to answer questions about their daily experiences, but if they missed a day, the language, “since you last completed this form,” may have caused confusion.

In conclusion, associations among within-person and between-person mindfulness and negative affect were observed concurrently on the same-day, whereas only between-person associations were observed for greater mindfulness prospectively predicting less negative affect. Most associations between mindfulness and loss-of-control were observed between-person, whereas no significant associations between negative affect and eating were observed at all. Together, findings highlight the role that interindividual variations in mindfulness may have on loss-of-control eating. Within future work, it will be important to replicate the results of this study among a larger and more diverse sample of adolescents and to investigate associations using EMA within a randomized controlled design.

## Supplementary Material

Figure S1: Variability in each outcome by participant

Additional supporting information can be found online in the Supporting Information section at the end of this article.

## Figures and Tables

**TABLE 1:** Descriptive statistics for participants.

	Percentage (*n*)
Gender	
Female	77% (35)
Male	23% (10)
Race/ethnicity	
Non-Hispanic White	72% (32)
Hispanic/Latino	20% (9)
Native American or Asian	8% (4)
BMI percentile	
95th percentile and above	42% (19)
85th–94th percentile	50% (22)
62nd–84th percentile	8% (4)
Endorsed at least one episode of	
Loss-of-control	38% (17)
Overeating	38% (17)
	***M (SD)***
Age, years	14.4 (1.74)
BMI	1.69 (.53)

**TABLE 2:** Descriptive statistics for key study variables.

	M^a^	SE^a^	Range	ICC
Mindfulness	4.46	.14	.67–6	.70
Negative affect	1.96	.08	1–4.6	.46
Loss-of-control^b^	.11	.03	-	.42
Control of eating	3.83	.14	1–5	.53
Overeating^b^	.08	.02	-	.11

a Calculated from mixed models to accurately account for clustering within person and varying numbers of observations per person.

b Loss-of-control and overeating were measured as the presence or absence. Variability in study can also be observed in Figure S1.

## Data Availability

Data sharing is not applicable to this article as no new data were created or analyzed in this study.
