# Supplementary material for: Daily mindfulness, negative affect, and eating behaviors in adolescents at risk for excess weight gain
Source: Int J Eat Disord. Author manuscript; Available in PMC 2025 Oct 7. (PMC12503394; doi:10.1002/eat.23981)
Supplement: Figure S1: Variability in each outcome by participant [file NIHMS2110541-supplement-Figure_S1__Variability_in_each_outcome_by_participant.docx]

Supplemental Figure 1

*Variabilty in Each Outcome by Participant*

| 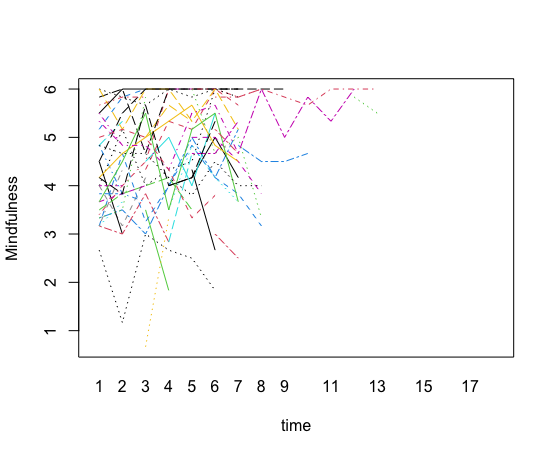 | 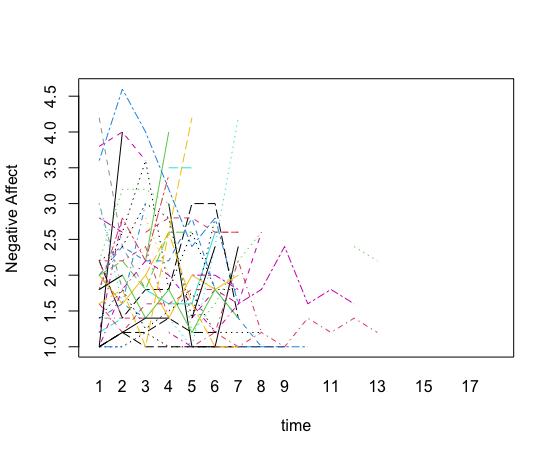 | 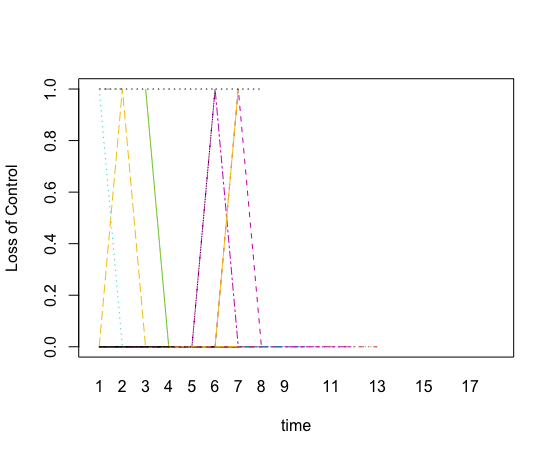 |
| --- | --- | --- |
| 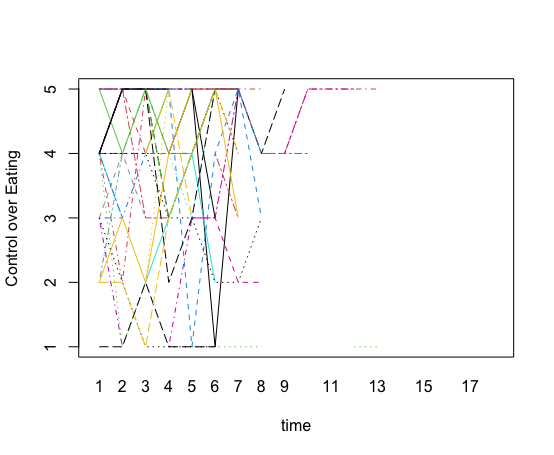 | 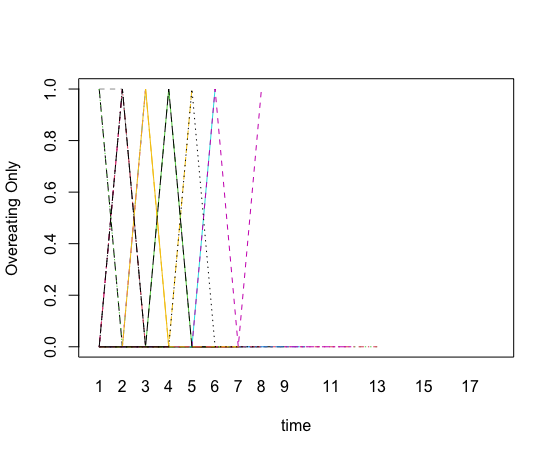 |  |

*Note.* This figure demonstrates the variability in mindfulness, negative affect, loss-of-control, control over eating, and overeating only over time by participant. Time refers to day in the study.
